# A Novel Human Ghrelin Variant (In1-Ghrelin) and Ghrelin-O-Acyltransferase Are Overexpressed in Breast Cancer: Potential Pathophysiological Relevance

**DOI:** 10.1371/journal.pone.0023302

**Published:** 2011-08-04

**Authors:** Manuel D. Gahete, José Córdoba-Chacón, Marta Hergueta-Redondo, Antonio J. Martínez-Fuentes, Rhonda D. Kineman, Gema Moreno-Bueno, Raúl M. Luque, Justo P. Castaño

**Affiliations:** 1 Department of Cell Biology, Physiology and Immunology, Instituto Maimónides de Investigación Biomédica de Córdoba (IMIBIC), University of Córdoba, Hospital Universitario Reina Sofía, and CIBERobn Fisiopatología de la Obesidad y la Nutrición, Córdoba, Spain; 2 Department of Biochemistry, Instituto de Investigaciones Biomédicas “Alberto Sols”, CSIC-UAM, Instituto de Investigación Sanitaria La Paz (IdiPAZ) and Fundación MD Anderson Internacional, Madrid, Spain; 3 Section of Endocrinology, Diabetes, and Metabolism, Department of Medicine, University of Illinois at Chicago, and Research and Development Division, Jesse Brown Veterans Affairs Medical Center, Chicago, Illinois, United States of America; University of Chicago, United States of America

## Abstract

The human ghrelin gene, which encodes the ghrelin and obestatin peptides, contains 5 exons (Ex), with Ex1-Ex4 encoding a 117 amino-acid (aa) preproprotein that is known to be processed to yield a 28-aa (ghrelin) and/or a 23-aa (obestatin) mature peptides, which possess biological activities in multiple tissues. However, the ghrelin gene also encodes additional peptides through alternative splicing or post-translational modifications. Indeed, we previously identified a spliced mRNA ghrelin variant in mouse (In2-ghrelin-variant), which is regulated in a tissue-dependent manner by metabolic status and may thus be of biological relevance. Here, we have characterized a new human ghrelin variant that contains Ex0-1, intron (In) 1, and Ex2 and lacks Ex3-4. This human In1-ghrelin variant would encode a new prepropeptide that conserves the first 12aa of native-ghrelin (including the Ser3-potential octanoylation site) but has a different C-terminal tail. Expression of In1-variant was detected in 22 human tissues and its levels were positively correlated with those of ghrelin-O-acyltransferase (GOAT; p = 0.0001) but not with native-ghrelin expression, suggesting that In1-ghrelin could be a primary substrate for GOAT in human tissues. Interestingly, levels of In1-ghrelin variant expression in breast cancer samples were 8-times higher than those of normal mammary tissue, and showed a strong correlation in breast tumors with GOAT (p = 0.0001), ghrelin receptor-type 1b (GHSR1b; p = 0.049) and cyclin-D3 (a cell-cycle inducer/proliferation marker; p = 0.009), but not with native-ghrelin or GHSR1a expression. Interestingly, In1-ghrelin variant overexpression increased basal proliferation of MDA-MB-231 breast cancer cells. Taken together, our results provide evidence that In1-ghrelin is a novel element of the ghrelin family with a potential pathophysiological role in breast cancer.

## Introduction

Ghrelin is a multifunctional 28-amino acid (aa) hormone mainly produced in the stomach [1], but also produced by a wide variety of tissues where it can act as a paracrine/autocrine factor [2]. Ghrelin can be acylated by the ghrelin O-acyltransferase (GOAT) enzyme [3], [4], to yield the natural ligand of the only known ghrelin receptor, the growth hormone (GH) secretagogue receptor type-1a (GHSR1a) [1]. To date, the acyl-ghrelin/GHSR1a system has been directly associated with multiple physiological functions related to regulation of energy balance and metabolic function at the central and peripheral level [5], [6]. However, ghrelin and its receptor are also present in many endocrine and non-endocrine tumor cell types (for example, gastroenteropancreatic, pituitary, prostate, breast), and in their related cancer cell lines, where ghrelin has been shown to control neoplastic cell proliferation [7]–[10]. Yet, the precise role of the ghrelin system in cancer is poorly understood. As an example, a truncated isoform of GHSR1a, the GHSR type 1b (GHSR1b), is found in the majority of the tumors and cancer cell lines cited above, however, its potential role in tumor regulation remains unknown [11], [12]. In fact, there is only isolated evidence that GHSR1b can act as a co-receptor with the neurotensin receptor 1 to form a novel receptor for neuromedin U in lung cancer [13].

Human ghrelin is encoded in the *GHRL* gene. Its transcription generates a 117-residue immature prepro-peptide (prepro-ghrelin), which can be acylated (or not) by GOAT and further processed by prohormone convertases (PC1/3 [14] and PC2 [15]), resulting in acyl-ghrelin (AG) or unacylated-ghrelin (UAG) [14], [16]. Original studies indicated that human *GHRL* spans 5 kb on chromosome 3, with a 20 bp non-translated Ex (termed Ex0) and four coding Ex (Ex1–4), where the prepro-ghrelin signal peptide is encoded by Ex1, and the coding sequence (CDS) of the mature-ghrelin hormone is encoded by Ex1 and Ex2 [14]. Yet, unexpectedly, recent studies demonstrated that prepro-ghrelin mRNA also encodes obestatin [17], a controversial peptide whose tissue- and cell-expression, and potential biological effects differs from those of ghrelin [18]–[20]. In addition to ghrelin and obestatin, other peptides could also be encoded by the human *GHRL*, as re-examination of its genomic structure revealed that this gene spans 7.2 kb, with a novel upstream Ex-1 and extended exonic regions of Ex0 and Ex1 [21]. Accordingly, multiple tissue-specific, alternatively spliced transcripts generated by the upstream exons have been recently identified, many of them lacking regions encoding ghrelin or obestatin and some encoding unique ghrelin/obestatin-derived transcripts, including Δex1-proghrelin, Δex1-2-proghrelin, Δex1-3-proghrelin [22]. Additionally, an alternative splice site in the intron 1 of the human and rodent ghrelin genes has been shown to result in translation of a biologically active peptide identical to mature ghrelin, except for the loss of a single glutamine residue at position14 (des-Gln14-ghrelin) [23]. Similarly, a ghrelin transcript lacking the exon that encodes obestatin (Ex3-deleted ghrelin) was identified and found to be highly expressed in human breast and prostate cancer tissues and derived-cell lines [7], [24], [25]. Hence, current evidence supports the notion that the human ghrelin gene is not simple, but a complex, multifarious system, regulated at multiple levels, which yields diverse transcripts and proteins with multiple functions, many of them likely still to be discovered.

In favor of this hypothesis, we recently described a murine ghrelin transcript that contains Ex2, In2 and Ex3, but lacks Ex1, Ex4 and Ex5, thus termed In2-ghrelin [26], whose mRNA levels were found to be highly expressed in the pituitary and hypothalamus as compared with native-ghrelin [26]. Of note, In2-ghrelin expression was regulated under severe metabolic conditions (fasting and obesity) [26], and paralleled strikingly those of GOAT mRNA [27], suggesting that In2-ghrelin can be a primary substrate for GOAT. The sequence of the retained mouse intron shows high homology with the human In1 [22], [28], and given that human intron 1 possesses the typical hallmarks of retained introns (i.e., to be short introns flanked by weak splice sites) [29], we postulated that intron 1 could also be retained in the human ghrelin gene and give rise to a similar ghrelin variant. Accordingly, in the present work, we aimed at investigating the potential existence of this variant in humans and, consequently, to compare and contrast the tissue distribution of this putative transcript with that of native ghrelin, and genes critically related to ghrelin peptide modification (GOAT) and function (GHSR1a and GHSR1b). Our results demonstrate the existence of such a new spliced mRNA variant of human GHRL, which retains In1, In1-ghrelin, and is present in a variety of human tissues and reveal an unexpected regulation of this system in human breast cancer.

## Materials and Methods

### Patients and samples

A commercial panel of total RNA from various human tissues was obtained from Clontech (Total Master Panel II and pituitary poly-A RNA; Palo Alto, CA), where each tissue sample is a pool of multiple individuals. A group of 40 sporadic invasive ductal breast carcinomas classified as high grade tumors (G3) and 4 normal breast samples obtained from the adjacent regions to the tumor area were obtained from Tumour Bank-CNIO (Madrid). Histological and immunohistochemical analysis classified the 40 samples as positive or negative carcinomas for estrogen receptor (ER), progesterone receptor (PR) and Her2neu, being 40%, 29% and 35% positive for ER, PR and Her2neu, respectively. Specifically, 29% of the tumors were classified as luminal, 35% as Her2neu and 36% as basal. The mean patient age at surgery was 53 years (range, 27–87 years). This study was approved by the Ethics Committee of the University of Cordoba, and the Tumour Bank-CNIO. Written informed consent was obtained from each patient before study entry.

### Cell lines

As previously reported [30], the MDA-MB-231 cell line (ATCC, Manassas, VA) was maintained in Dulbecco's Modified Eagle Medium (DMEM) supplemented with 10% fetal bovine serum (FBS), 1% antibiotic-antimycotic (100X solution: 10,000u penicillin, 10,000 µg streptomycin, and 25 µg amphotericin B/ml, Invitrogen) and 2 mM L-glutamine at 37°C and 5% CO_2_. For *in vitro* treatments, native MDA-MB-231 cells were cultured during 24 h with DMEM complemented with charcoal-treated serum and were challenged with human ghrelin, human UAG, β-estradiol or tamoxifen (10^−7^ M for every compound, Sigma), or were incubated with vehicle (Controls).

### RNA isolation and reverse transcription (RT)

Nucleic acids were isolated with Trizol (Invitrogen, Barcelona, Spain) following the manufacturer's instructions and treated with DNase as previously described [31], [32]. The amount of RNA recovered was determined using the Ribogreen-RNA quantification kit (Molecular Probes, Eugene, OR, USA). Total RNA (2 µg) was reverse transcribed (RT) with the cDNA First-Strand Synthesis kit using random primers according to the manufacturer's instructions (Fermentas, Hanover, MD, USA).

### Primer selection and standard or quantitative real- time RT-PCR (qrtRT-PCR)

Primers used for standard-PCR and qrtRT-PCR (tables S1 and S2) were selected using the human ghrelin gene sequence as template (GeneID: 51738) and primer3-software (http://frodo.wi.mit.edu). For standard RT-PCR, 2X Master Mix PCR reagent (MRI-Fermentas) and brain cDNA were used following the manufacturer's recommendations. The thermocycling profile consisted of one cycle of 94°C (4 min), followed by 30–40 cycles of 94°C (30 sec), 58–62°C (depending on the primer set; 30 sec), and 72° (30–60 s; depending on the product size), and a final cycle at 72°C (5 min). Amplifications were performed using the iCycler system (Biorad, CA, USA). Absolute expression levels of ghrelin, In1-ghrelin variant, GOAT, GHSR1a, GHSR1b, Ki-67 and Cyclin D3 were screened by qrtRT-PCR using specific primers (table S2). Details regarding selection of primers, verification of primer specificity, confirmation of primer efficiency, construction of standard curves for each transcript as well as the details of the development, validation, and application of a qrtRT-PCR to measure expression levels of different human genes have been reported previously [33]. The final volume of the PCR reaction was 25 µl including 100 ng of sample and 12,5 µl of IQ™ SYBR Green Supermix (Biorad). The thermocycling profile consisted of 40 cycles at 94°C (30 s), 61°C (30 s), and 72°C (30 s). Amplifications were performed using the iCycler IQ™ system (Biorad). To determine the starting copy number of cDNA, we used a specific standard curve of each transcript run in the same plate. Non-RT RNA samples and non-DNA controls were run on each plate to control for genomic DNA or exogenous contamination, respectively. Final PCR products were subjected to graded temperature-dependent dissociation to verify that only one product was amplified and then run on agarose gels to confirm that only one band, of the expected size, were amplified. All PCR products were then column-purified (Bioneer Inc., CA, USA) and sequenced to confirm target specificity. To control for variations in the amount of RNA used, the expression level of one housekeeping gene (beta-actin) was determined for each sample where beta-actin copy number was not altered between experimental groups (data not shown).

### Identification of the human and baboon In1-ghrelin variant

**Figure 1:**
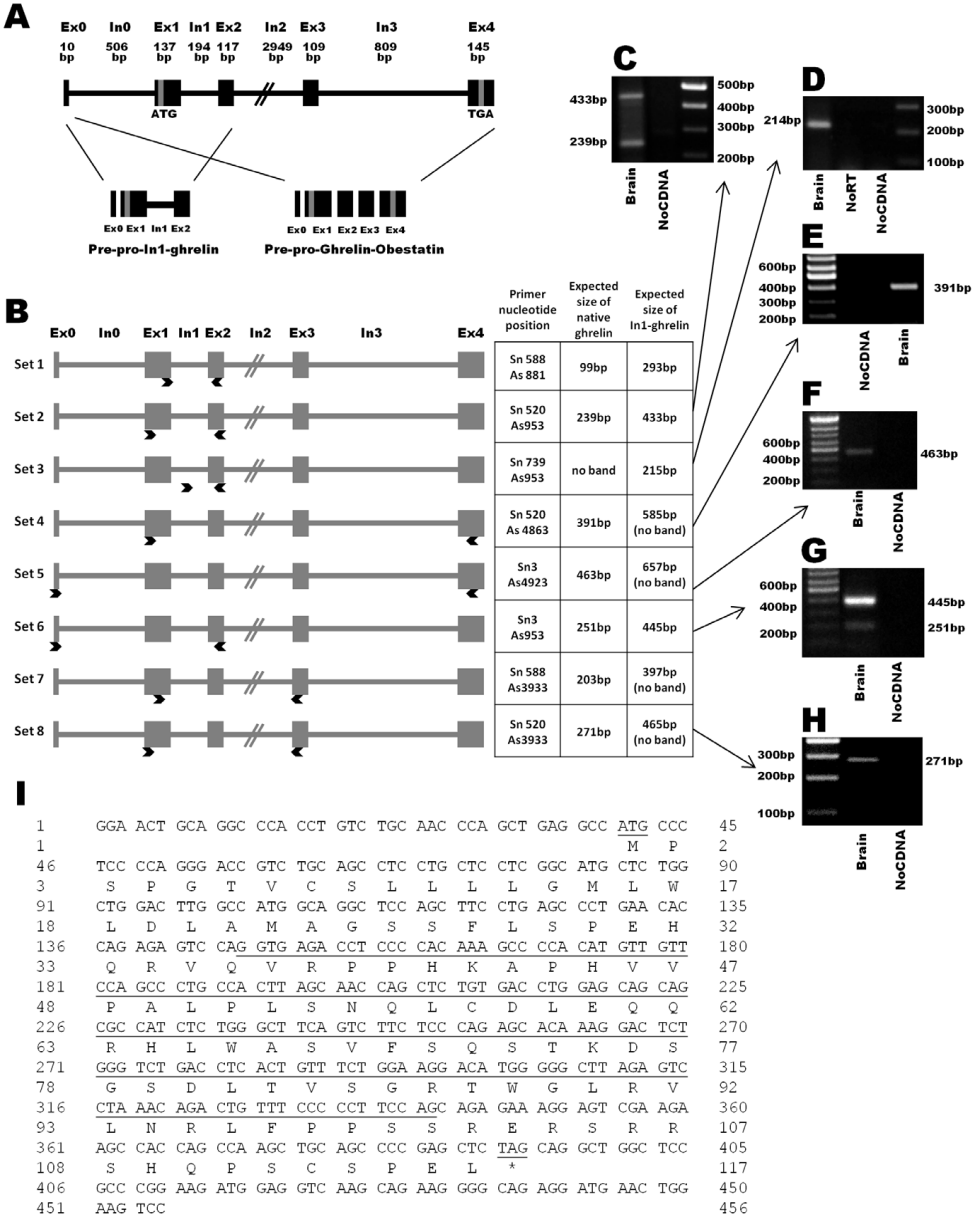
Schematic representation of the human In1-ghrelin variant. (A) Classical organization of human ghrelin/obestatin gene consisting of 5 exons (Ex0–4) and 4 introns (In0–3). Structure of prepro-ghrelin/obestatin and prepro-In1-ghrelin mRNA precursors is also indicated. (B) Schematic diagram showing the primer position (indicated with arrows) used to detect, clone and/or quantitatively amplify the human In1-ghrelin. Exons are represented as boxes and introns as lines. (C–H) PCR products obtained using the previously presented set of primers using brain tissues as template, separated on agarose gel and stained with ethidium bromide. In addition, non-RT RNA samples and non-DNA controls were run to control for genomic DNA or exogenous contamination, respectively. (I) Nucleotide and amino acid sequences of In1-ghrelin. In1 sequence and the putative start and stop codons are underlined. Exon:Ex, Intron:In, Base pair:bp.

In order to search for a putative In1-ghrelin variant in the human gene, we designed several sets of PCR primers located in different exons and introns of the human *GHRL* gene (Fig. 1B). PCRs were done as described above. The novel sequence of the In1-ghrelin variant was submitted to Genbank (accession #: GU942497). In the case of the identification of the In1-ghrelin variant in the primate model olive baboon (*Papio anubis*) (Fig. S1) we used a similar strategy to that explained for human but with primers designed using *Macaca mulata, Pan troglodytes* and human ghrelin gene sequences as these genomes are highly similar. The novel sequence of the baboon In1-ghrelin variant was submitted to Genbank (accession #: HM048926).

Comparisons of human and baboon In1-ghrelin variants and mouse In2-ghrelin variant at the mRNA level and at the level of the predicted protein were done with BioEdit 5.0 software. Prediction of prepro-peptide cleavage sites was done with a specific software (ProP-1.0 Server).

### Cloning of In1-ghrelin variant and transfection of breast cancer cell lines

PCR amplification of the full coding sequence of In1-ghrelin variant was accomplished using cDNA of MDA-MB-231 cells as template, Sn-520 and As-953 primers (table S1), and the high-fidelity-polymerase i-MAXII (iNtRON Biotechnology, Seongnam, Korea). The PCR product was directly cloned into *pGEM-T* vector (Promega, Madrid, Spain) to be further subcloned into the *pCDNA3.1+* vector (Invitrogen) using a specific set of primers with the HindIII and BamHI restriction sites, respectively (In1-Hind-Up: TCTCAAGCTTATGCCCTCCCCAGGGAC and In1-Bam-Low: TGTGGGATCCCTAGAGCTCGGGGCTGCAG). MDA-MB-231 cells were transfected using Lipofectamine-2000™ (Gibco, Barcelona, Spain) as previously reported [31], [34] to obtain In1-ghrelin variant overexpressing MD-MB-231 cells. Specifically, 0.5 ug of In1-ghrelin variant-*pCDNA3.1+* plasmid were used to transfect MDA-MB-231 cells plated on 6-wells plates. MDA-MB-231 cells transiently transfected with empty *pCDNA3.1+* (mock transfected) were used as negative control.

### Western blotting

In order to detect the human In1-ghrelin variant protein, we searched for commercially available antibodies raised against the region that native ghrelin shares with In1-ghrelin variant (12 first amino acids of mature native ghrelin). We found three antibodies with this characteristic, two of them designed against acyl-ghrelin (Rabbit Anti-Human-Ghrelin, Alpha Diagnostic, San Antonio, TX (Cat. # GHS11-S) and Chicken Anti-Human-Ghrelin, GeneTex, Inc., Irvine, CA (Cat. # GTX15861, USA)] and a third one against unacyl-ghrelin (Anti-GHRL (ab1), Sigma). We have performed western blotting with the three antibodies using a number of different conditions (nitrocellulose or PVDF membranes, blocking with BSA o milk, different blotting times, different concentrations of primary and secondary antibodies) and protein obtained from various ghrelin- and In1-ghrelin-expressing cell lines. However, all these attempts were unsuccessful, as we did not find immunoreactivity against any endogenous peptide (either native ghrelin or In1-ghrelin variant).

### *In Vitro* Proliferation Assays

Basal cell proliferation was evaluated in mock- or In1-ghrelin variant transiently transfected MDA-MB-231 cells using Alamar-Blue reagent (Biosource International) as previously reported using other transcripts [31], [34]. Briefly, after transfection, 2000 mock- or In1-ghrelin variant transfected cells/well were seeded in a 96-well plate and cultured during 5d. Then, cells were starved (serum-free-medium) for 24 h and cellular proliferation rate was measured, every 24 h, for the following 4 days. The day of measurement, cells were incubated for 3 h in 10% alamar blue/DMEM with FBS (10%) and then, alamar reduction was measured in a BioTek-Synergy-HT fluorescence plate reader (BioTek-Instruments, Inc., Vermont, USA), exciting at 560 nm and reading at 590 nm. Medium with alamar blue was replaced and washed twice with fresh medium immediately after each measurement. Kinetics curves were fitted to exponential growth curves with GraphPad-Prism4 (GraphPad-Software, San Diego, CA). Doubling time and K growing constant were calculated from these exponential curves and the results obtained in MDA-MB-231 cells transiently transfected with the In1-ghrelin variant were expressed as percentage of control (mock-transfected cells). In all instances, cells were seeded in quadruplicate and the assay was repeated three times with independent transfections.

### Statistical analysis

**Figure 2:**
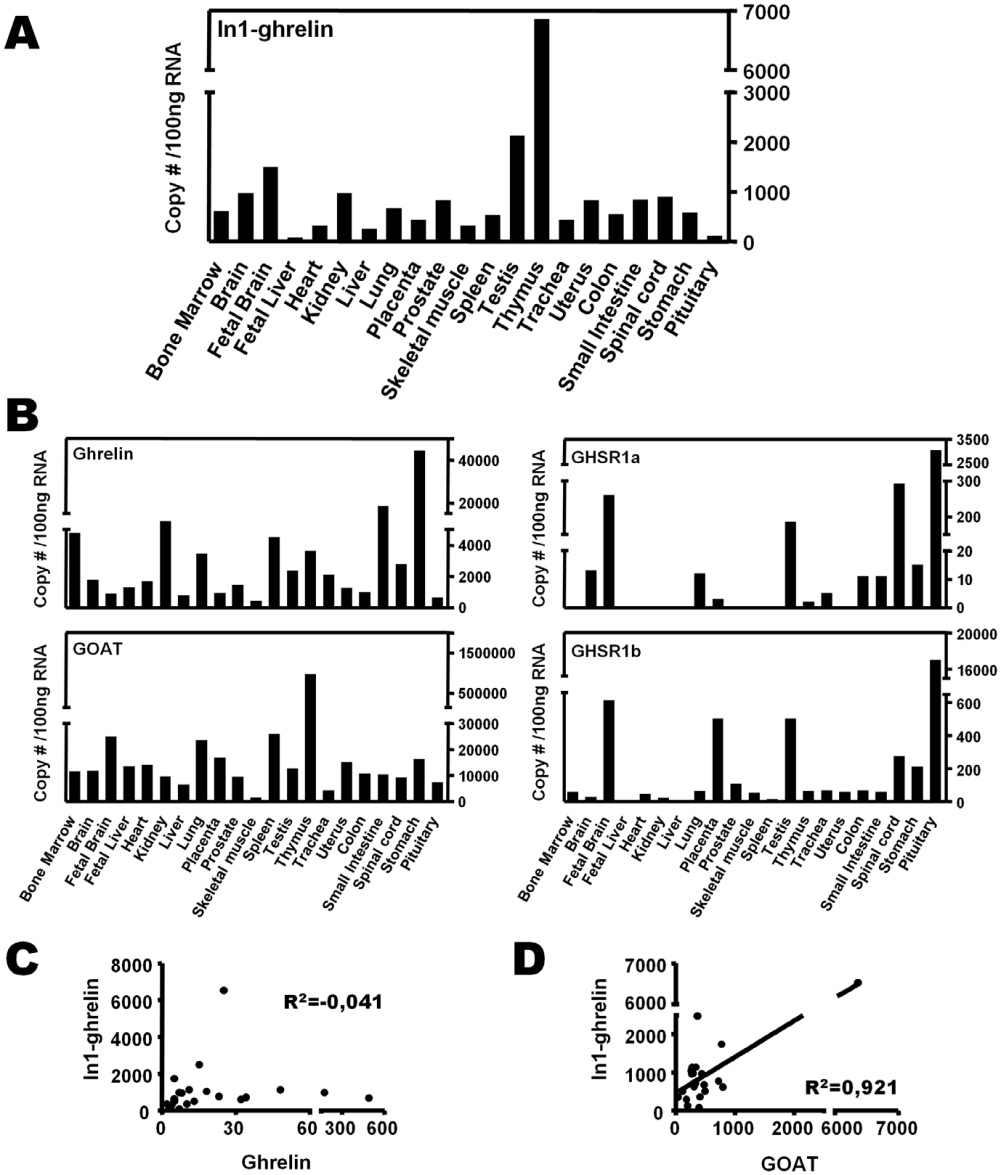
Tissue distribution of the human In1-ghrelin variant. (A) Quantitative (qrtPCR) expression levels of In1-ghrelin using a commercial panel of total RNA from various human tissues obtained from Clontech, where each tissue sample is a pool of multiple individuals and represents a mean expression level in this particular tissue (only a single sample of each tissue is provided). (B) Quantitative (qrtPCR) expression levels of ghrelin, GOAT, GHSR1a and GHSR1b using the same commercial panel of total RNA from various human tissues obtained from Clontech. (C) Correlation between In1-ghrelin and native ghrelin expression in human tissues (G) Correlation between In1-ghrelin and GOAT expression in human tissues.

**Figure 3:**
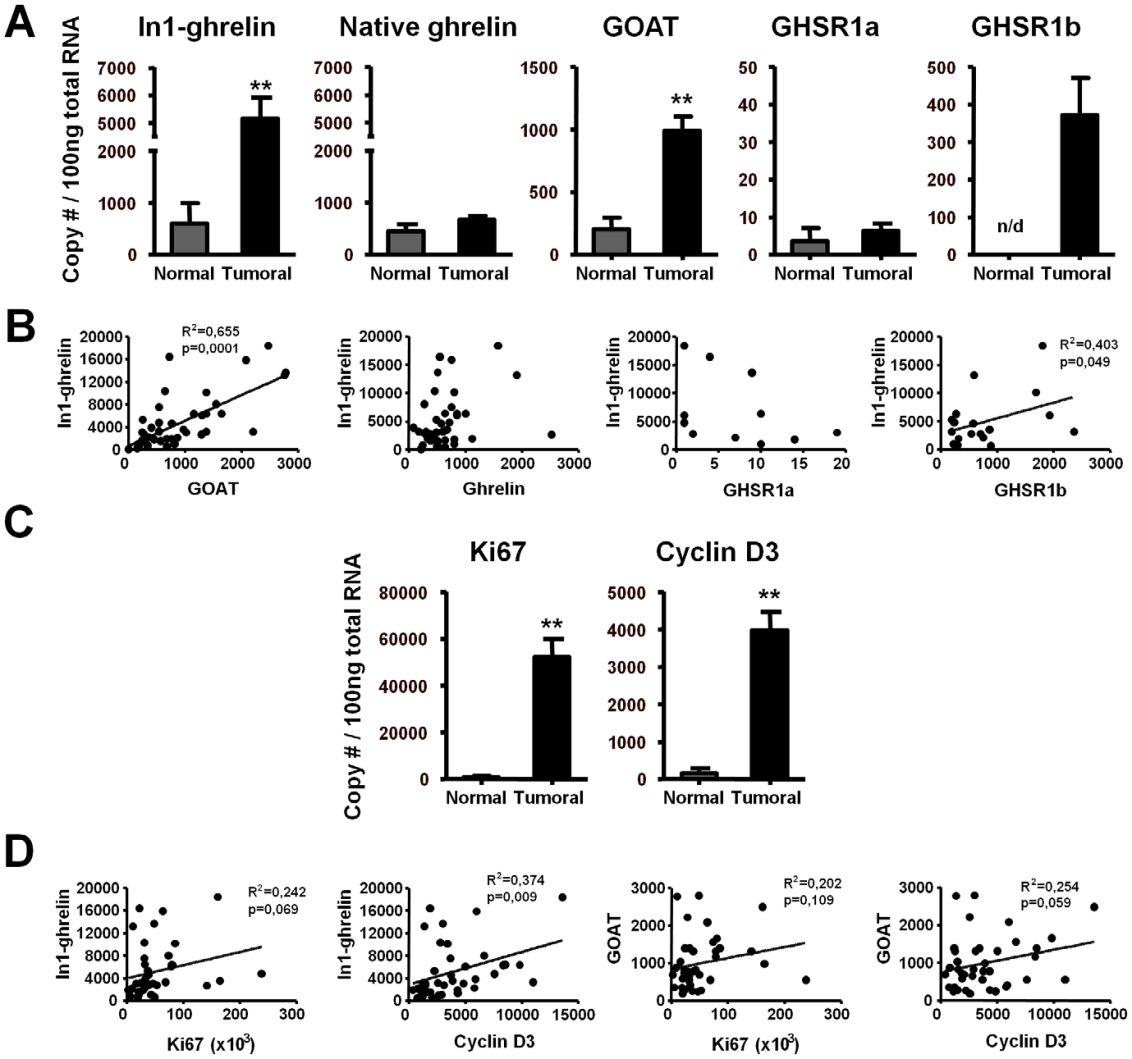
In1-ghrelin variant in human breast samples. (A) Quantitative expression levels of the ghrelin-axis in normal (n = 4) and breast cancer samples (n = 40). (B) Correlations between In1-ghrelin and ghrelin, GOAT, GHSR1a/1b expression in breast cancer samples. (C) Quantitative expression levels of Ki67 and cyclin-D3 in normal and breast cancer samples. (D) Correlation values between In1-ghrelin and GOAT with Ki67 or cyclin-D3 expression in breast cancer samples. Values are shown as the mean ± S.E.M (** P<0.01; indicate differences from controls).

Samples from all patients, tissues or cell cultures within the same experiment were processed at the same time and therefore correlations between expression of transcripts in different tissues (Fig. 2 and 3) were assessed by one-tailed Pearsońs correlation test while variations between normal vs. tumoral breast samples (Fig. 3) were assessed by Students t-test. Effect of pCDNA (mock) or In1-ghrelin variant transfection on *in vitro* proliferation assays was assessed by two-way-ANOVA followed by a Newman-Keuls test for multiple comparisons. P≤0.05 was considered significant. All values are expressed as means ± SEM.

## Results

### Identification of the human In1-ghrelin variant using standard RT-PCR

As illustrated in Fig. 1A, the transcript region of the human ghrelin gene (*GHRL*) consists of one noncoding (Ex0) and four coding (Ex1-Ex4) exons, where the 28-aa mature ghrelin is encoded by Ex1-Ex2, while the 23-aa mature obestatin is encoded by Ex3. In order to search for a putative In1-ghrelin variant in the human gene, we designed several sets of PCR primers located in Ex1 (sense) and Ex2 (antisense), thereby flanking In1 of the human *GHRL* gene (Fig. 1B, sets1-2). Using brain cDNA as a template, two PCR bands were amplified by each primer set, one of the expected size for native-ghrelin (99bp and 239bp, respectively) and one for the putative In1-ghrelin variant (293bp and 433bp, respectively) (Fig. 1B–C). Sequencing of the PCR products confirmed that the smaller products (99 and 293bp) corresponded to native-ghrelin, while the longer ones (239 and 433bp) corresponded to a variant that retains the entire In1. Additionally, we designed a pair of primers that would exclusively amplify the In1-variant using a sense primer located entirely in the intronic region (Fig. 1B, set-3), thus resulting in a single band of the expected size and sequence (215bp; Fig. 1D). In contrast, no bands were amplified in non-reverse transcribed and non-cDNA control, demonstrating that the band observed was not the consequence of genomic or external contamination, respectively. In order to study if other exons, different to Ex1-2, are present in the novel human In1-ghrelin mRNA variant, we selected primers located in Ex0, 3, and 4 (Fig. 1B, sets4–8 and Fig. 1E–H) and combined these with primers in Ex1 and 2. PCR products obtained with these combinations of primers revealed that human In1-ghrelin variant comprises Ex0, Ex1, In1 and Ex2, but lacks Ex3 and Ex4. This novel sequence of the In1-ghrelin variant was submitted to Genbank (accession #: GU942497).

Since In1-variant and native-ghrelin share the Ex0 and 1, it seems reasonable to assume that the human In1-ghrelin variant shares the same start codon as native-ghrelin, located in the Ex1. Assuming that translation of In1-ghrelin variant mRNA would encode a 117aa protein (prepro-In1-ghrelin), sharing the first 36aa of native-prepro-ghrelin, including the signal peptide (24aa: MPSPGTVCSLLLLGMLWLDLAMA) and the first 12aa of the mature native-ghrelin, which includes the putative acylation site at Ser3. However, as is the case of the mouse In2-ghrelin variant [26], the reading frame of the human In1-ghrelin variant would be altered by the retention of intron 1, thereby encoding a completely novel C-terminal tail, where the obestatin would not be produced. Interestingly, we have also identified the In1-ghrelin variant in a primate model (Olive baboon, *Papio anubis*) (Fig. S1) using a similar strategy to that explained for human but with primers designed using *Macaca mulata, Pan troglodytes* and human ghrelin gene sequences, as these genomes are highly similar. Comparisons of human and baboon In1-ghrelin and mouse In2-ghrelin variants show high inter-specific homology (at the mRNA level and at the level of the predicted protein) (Fig. S1).

Using a specific software to predict prepro-peptide cleavage sites (ProP-1.0 Server), we identified various putative cleavage sites (for example, RH (63/64) and KA (42/43)) located in the human prepro-In1-ghrelin, which exhibit a similar cleavage score to the cleavage sites located in the prepro-ghrelin/obestatin precursor, whose processing generates ghrelin and obestatin. Although, it would be predicted that the first 12aa of native-ghrelin would be retained in a peptide encoded by In1-ghrelin transcript, use of three commercially available antibodies directed against this epitope [Rabbit Anti-Human-Ghrelin, Alpha Diagnostic, San Antonio, TX (Cat. # GHS11-S), Chicken Anti-Human-Ghrelin, GeneTex, Inc., Irvine, CA (Cat. # GTX15861, USA) and Anti-GHRL (ab1), Sigma] failed to detect either native ghrelin or In1-ghrelin proteins by western (data not shown).

### mRNA expression levels of In1-ghrelin variant in human tissues

In order to analyze the presence and abundance of In1-ghrelin variant in human tissues, we used the primer set 3 shown in Fig. 1B, and table S2 in qrtRT-PCR to exclusively amplify this transcript. As template, we used a commercial panel of human tissues, where each sample (tissue) is a pooling of several samples coming from various individuals, and therefore represents an average expression level in this particular tissue. Results of qrtRT-PCR showed that In1-ghrelin variant was expressed in the 22 tissues analyzed. However, the expression levels were quite variable (Fig. 2A), with In1-ghrelin variant mRNA being abundantly expressed in thymus, testis, kidney, stomach, uterus or brain (as previously reported [35]). We also measured the expression levels of native-ghrelin, GOAT, GHSR1a and GHSR1b, in the same human samples and the results are shown in Fig. 2B and table S3. Interestingly, we observed that expression levels of native-ghrelin and In1-ghrelin variant did not share a common tissue-specific expression pattern (R^2^ = −0,041) (Fig. 2C). In contrast, when comparing the expression levels of each of the ghrelin-related transcript to GOAT, we found a strong correlation between mRNA levels of In1-ghrelin variant and GOAT (R^2^ = 0,921; Fig. 2D) in all the tissues analyzed, while there was no significant correlation between native-ghrelin and GOAT (R^2^ = −0,025). Finally, our data showed that mRNA expression levels of both ghrelin receptors (GHSR1a and R1b) did not correlate with native or In1-variant ghrelin in the human tissues analyzed (data not shown).

### In1-ghrelin variant is overexpressed in breast cancer samples

qrtRT-PCR results showed that In1-ghrelin variant is expressed in normal mammary glands and, surprisingly, the expression levels of In1-ghrelin variant were found to be 8-fold up-regulated in a series of 40 sporadic invasive ductal breast carcinomas classified as high grade tumors (G3) compared with control samples (p = 0,0042) (Fig. 3A; table S4). The expression of In1-ghrelin variant was not correlated with the presence of ER or PR in this series but showed a trend to be correlated with Her2neu presence, so that the expression level of In1-ghrelin is numerically, albeit non-significantly (p = 0.075), higher in Her2neu tumors compared with luminal and basal tumors (data not shown).

Similarly, the mRNA levels of GOAT were up-regulated in breast cancer samples (Fig. 3A), and were significantly correlated with the expression levels of In1-ghrelin variant (R^2^ = 0.655; p≤0.001; Fig. 3B). In striking contrast, expression levels of native-ghrelin did not differ between normal mammary gland and breast cancer samples (Fig. 3A; table S4). Likewise, mRNA levels of GHSR1a were low in both normal and tumoral breast samples (Fig. 3A; table S4), and no significant differences were found between those experimental groups. Interestingly, while expression of GHSR1b was not detected in normal mammary gland samples, it was highly expressed in breast cancer samples (Fig. 3A; table S4). Moreover, whereas GHSR1a mRNA levels do not correlate with native-ghrelin or In1-ghrelin variant levels (Fig. 3B), we found that expression levels of GHSR1b parallel those of In1-ghrelin (R^2^ = 0.403; p = 0.049; Fig. 3B) but not those of native-ghrelin (data not shown). As expected, the expression levels of two key tumoral markers of cell proliferation, Ki67 and cyclin-D3, were highly up-regulated in breast cancer samples as compared to their matched controls (p<0.01; Fig. 3C). Interestingly, the levels of expression of In1-ghrelin variant and GOAT levels showed a trend to or were positively correlated with the expression of Ki67 (p = 0.069 and p = 0.109, respectively) and cyclin-D3 (p = 0.009 and p = 0.059, respectively) in breast cancer samples (Fig. 3D).

### In1-ghrelin variant increases the proliferation rate of MDA-MB-231 cells and its expression is regulated by ghrelin and tamoxifen

**Figure 4:**
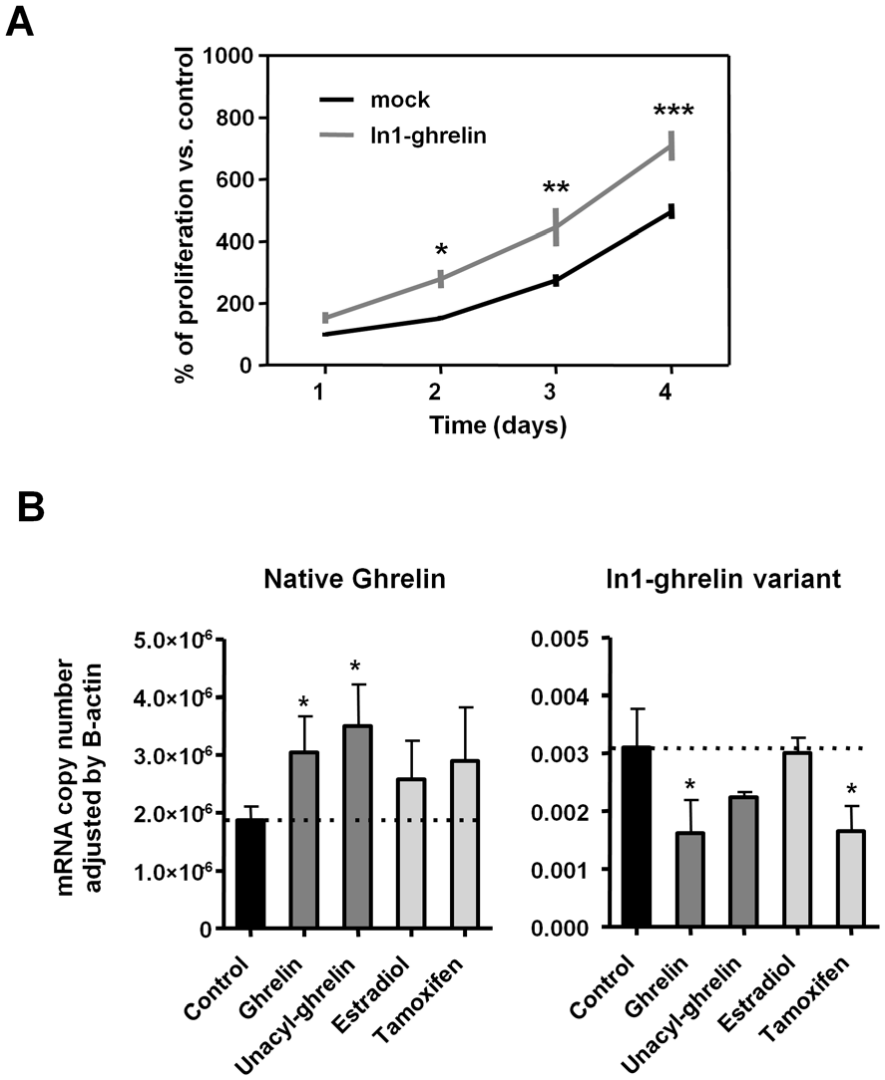
Effect and expression regulation of In1-ghrelin variant in MDA-MB-231 cells *in vitro*. (A) Proliferation kinetics of MDA-MB-231 transfected with In1-ghrelin or control-mock. (B) Regulation of native-ghrelin and In1-ghrelin expression in MDA-MB-231. The data represent the means ± SEM. Asterisks (* P<0.05, ** P<0.01, *** P<0.001) indicate differences from corresponding controls.

To investigate the functional consequences of In1-ghrelin variant expression and the regulation of In1-ghrelin transcript, we employed an *in vitro* approach using a breast cancer cell line, MDA-MB-231, which expresses In1-ghrelin variant, GOAT and GHSR1b transcripts at high levels while virtually lack the expression of native-ghrelin and GHSR1a (table S4), a pattern that is reminiscent of that observed in primary breast cancers. *In vitro* kinetic proliferation assays using MDA-MB-231 cells transfected with In1-ghrelin variant or empty vector (mock-control) indicated that cells over-expressing In1-ghrelin variant have a higher proliferative rate compared with mock-transfected cells (Fig. 4A). Specifically, cells that over-express In1-ghrelin variants exhibited a shorter doubling time than mock-transfected cells (1.21 days vs. 1.43 days, respectively) these differences being progressively more significant at 2, 3, and 4 days after transfection (p≤0.05, p≤0.01, and p≤0.001, respectively). Of note, expression levels of the native-ghrelin and In1-ghrelin variant transcripts were divergently regulated, in that treatment (24 h) with AG or UAG increased native-ghrelin mRNA levels, while In1-ghrelin variant levels were reduced, although the negative impact of UAG did not reach statistical significance (Fig. 4B). Tamoxifen treatment tended to increase native-ghrelin expression, whereas it significantly reduced In1-ghrelin variant mRNA levels. Conversely, estradiol (24 h) did not alter the expression levels of native-ghrelin or In1-ghrelin variant (Fig. 4B). It should be noted that expression levels of GOAT, GHSR1a and GHSR1b were not significantly altered in MDA-MB-231 with these treatments (data not shown).

## Discussion

Results of the current study confirm, as indicated previously in Gahete et al. [35], and, for the first time demonstrate, that the human ghrelin gene, similar to that reported in the mouse gene [26], can retain In1, one type of alternative splicing likely associated with the failure of intron detection which occurs more frequently in short introns flanked by weak splice sites [29]. Specifically, human In1-ghrelin variant contains Ex0, Ex1, In1 and Ex2 but lacks Ex3 and Ex4. Based on the fact that the In1-variant and native-ghrelin share the Ex0 and 1, we postulate that both share the same start codon, located in the Ex1. In this case, the first 36aa of the prepro-In1-ghrelin, including the signal peptide, and the first 12aa of the mature native-ghrelin, including the putative acylation site at Ser3 and the residues found to be necessary for acylation (Gly1 and Phe4) [4], would be identical to that encoded by the native-prepro-ghrelin mRNA. However, as occurred with the orthologous mouse In2-ghrelin variant, the reading frame of the human In1-ghrelin variant would be altered by the intron retention, thus encoding a completely novel C-terminal tail where obestatin would not be produced. In particular, the novel prepro-In1-ghrelin would possess a C-terminal tail with various putative cleavage sites, which exhibit a similar cleavage score to the cleavage sites located in the prepro-ghrelin/obestatin precursor whose processing generates ghrelin and obestatin, suggesting that different peptides could be processed from the In1-ghrelin variant. Interestingly, baboon ghrelin gene also undergoes processes of In1-retention which, coupled to the fact that In1-ghrelin variants are highly conserved across species (human, baboon and mouse) suggest that relevant functional parallels of this novel transcript may have occurred through mammalian evolution.

There is increasing evidence that the *GHRL* gene is very complex and can be regulated at multiple levels [22], [36]. In fact, several peptides are generated by alternative splicing of the ghrelin gene or by post-translational modifications [14], [16], [21], [37]. Our data demonstrate that an In1-ghrelin variant transcript is processed from the *GHRL* gene, similar to the In2-ghrelin variant previously reported in the mouse [26], and it is expressed in a wide variety of human tissues, suggesting this novel variant could be physiologically relevant and exert important biological actions in humans. Interestingly, expression levels of human native-ghrelin and In1-ghrelin variant were not correlated in the normal tissues studied, indicating that the production of both variants derived from the same gene could be differentially regulated in human tissues. Indeed, splice site recognition depends on several factors, such as mRNA regulatory elements or protein factors [38], which could modulate the expression of intron retaining and intron spliced isoform in a tissue-dependent manner [39], [40]. Importantly, we observed that expression levels of In1-ghrelin, but not of native-ghrelin, were strongly correlated with GOAT mRNA levels in human tissues. Because translation of In1-ghrelin variant would be likely to generate a prepro-peptide containing the same first 12aa than native-ghrelin, including the target Serine-3 octanoylated by GOAT, and the residues found to be necessary for its acylation (Gly1 and Phe4), it is reasonable to suggest that In1-ghrelin variant may be a primary substrate for GOAT in humans. This notion is supported by our recent results showing that mouse In2-ghrelin variant may also be a primary substrate for GOAT in mice [27], since its expression clearly parallels changes in the expression of GOAT in the pituitary of several mouse models analyzed (for example, fasting, obesity, knockout models). Thus, future studies should aim to unequivocally prove if the putative novel protein of the human In1-ghrelin variant can be acylated by GOAT.

Of note, our results revealed that In1-ghrelin variant may play a relevant role in human breast cancer. Indeed, In1-ghrelin variant expression was strongly up-regulated (8-fold) in ductal breast cancer samples compared with normal breast tissue. In line with this, a previous high-throughput sequencing analysis using tissue samples obtained from excised human breast tumors [41] identified an ORF expressed sequence tags (EST) (GenBank: BF929001.1) that shares 99% nucleotide sequence homology with the human In1-ghrelin variant identified herein. Since the study of Dias-Neto et al. focused on central portions of expressed coding regions by PCR amplifications, the clone homologous to In1-ghrelin variant (BF929001.1) contains the complete In1 of human ghrelin gene, but not the complete 5′- and 3′-regions. These results strongly suggest that the partial sequence obtained by Dias-Neto [41] corresponds to the novel human In1-ghrelin variant reported herein.

This report is also the first to show that the GOAT enzyme is strikingly overexpressed in breast cancer tissues compared with normal human mammary gland. Similar to that observed for normal tissues, absolute copy numbers of In1-ghrelin, but not of native-ghrelin, show a marked, positive correlation with GOAT expression in breast cancer, reinforcing the contention that In1-ghrelin variant represents the primary substrate for GOAT enzyme in this pathology. Furthermore, our results also unveiled a remarkable upregulation of GHSR1b, which positively correlated with In1-ghrelin expression, while GHSR1a and native-ghrelin were not altered in tumor samples. This latter finding is consistent with previous immunohistochemistry results showing very low levels of native-ghrelin in the normal breast epithelium, with moderate elevation in breast cancer samples [7]. The potential pathophysiological role of GHSR1b overexpression is both unknown and intriguing; although this truncated receptor can act as a dominant negative, by binding and internalizing GHSR1a [42], the disproportionately high levels of GHSR1b relative to GHSR1a (60-fold) found in human breast cancer samples suggest this receptor may serve a different role in this pathology. In line with this, GHSR1b has been shown to acts as a neuromedin U receptor in lung cancer by heterodimerizing with neurotensin receptor 1 [13]. Nonetheless, our current data may offer important clinical information since it provides primary evidence for the differential expression of In1-ghrelin, GOAT and GHSR1b in breast cancer tissues, which may represent novel diagnostic/prognostic markers and therapeutic targets for this pathology.

Human breast cancer MDA-MB-231 cells represent a suitable model to study In1-ghrelin function, as their expression patterns of the ghrelin-axis mimic those of breast cancer samples. Interestingly, expression of native-ghrelin and In1-ghrelin were oppositely regulated by AG and UAG in this cell model, providing, to our knowledge, the first evidence that ghrelin (AG and UAG) can regulate its own synthesis (native-ghrelin and In1-ghrelin transcripts). Thus, both AG and UAG increased the markedly low levels of expression of native-ghrelin in MDA-MB-231 cells, while decreasing the high levels of In1-ghrelin mRNA in these cancer cells, suggesting the existence of an auto-regulatory loop in this cell type. Most importantly, tamoxifen was also able to reduce In1-ghrelin mRNA levels while tended to increase native-ghrelin expression in MDA-MB-231 cells, an observation with potential patho-physiological implications since tamoxifen is currently a primary line of therapy for the treatment of estrogen receptor (ER) positive breast cancers and the ghrelin axis has been reported to exert relevant actions in cancer cells [7]. Although tamoxifen is an ER blocker and MDA-MB-231 is an estrogen-independent breast cancer cell line, several studies have reported effects of tamoxifen and other ER modulators such as raloxifene in ER-negative breast cancer cell lines via activation of several intracellular signaling pathways (protein kinase C, phospholipase D) through ER-independent mechanisms [43], [44].

An additional line of support for a patho-physiologically relevant role of In1-ghrelin in breast cancer arises from the positive association in breast cancer samples between In1-ghrelin expression and Ki67 and cyclin-D3, two markers linked to proliferation and ductal breast tumor grade [45]. Nevertheless, we should introduce the caveat that despite showing a strong significant correlation, this does not necessarily indicates a causal relationship for these two parameters. However, the plausible association between In1-ghrelin expression and increased proliferation is supported by our observation that In1-ghrelin overexpression in MDA-MB-231 cells causes a robust increase in proliferation rate, suggesting that blockade of In1-ghrelin variant may have some therapeutic benefit.

In conclusion, our current results provide novel evidence supporting that, as suggested by previous studies, the ghrelin system is functionally present in cancer cells [7], [9], . Furthermore, by demonstrating the relevant overexpression of novel components of the ghrelin system in this pathology, specifically the newly identified In1-ghrelin variant but also GOAT and GHSR1b, our study provides novel avenues to investigate the precise pathophysiological role and potential clinical implications of this family in breast cancer.

## Supporting Information

Figure S1 **mRNA and putative protein sequence comparison of human (Homo sapiens) and baboon (Papio anubis) In1-ghrelin variants, and the orthologous mouse (Mus musculus) In2-ghrelin variants.** (A) Alignment of the mRNA coding region (CDS) sequences of the In1-ghrelin and In2-ghrelin vaiants. Nucleotide sequences of the Exon(Ex) 1 of the In1-ghrelin and Ex2 of the In2-ghrelin variants that are shared with native-ghrelin are shown in boxes. CDS of baboon In1-ghrelin and mouse In2-ghrelin variants are shorter than human CDS because their mRNAs contain a stop codon (TAG; represented in shaded grey) located in the sequence of In1 or In2, respectively, while the stop codon of the human In1-ghrelin variant is located inside the Ex2 sequence. Therefore, human In1-ghrelin variant mRNA is not fully represented (nucleotides from 172 to 348 are not represented). Asterisks (*) indicate conserved nucleotides across species, which represent a 72% homology among human, baboon and mouse sequences. Of note, the homology between human and baboon In1-ghrelin variant mRNAs is 96%. (B) Alignment of putative In1-ghrein and In2-ghrelin variants protein sequences. Similar to that shown with nucleotide CDS sequences, human In1-ghrelin variant amino acid (aa) sequece is not completely represented (aa from 56 to 116 are not represented). The first 24 aa represents the signal peptide sequences in the three species. Asterisks (*) indicate conserved aa across species (16/24; 67% homology) while (#) indicate aa belonging to the same group (7/24) which results in a 96% homology (23/24 aa) among the human, baboon and mouse sequences. Boxes represent the aa sequence of In1-ghrelin and In2-ghrelin variants shared with native ghrelin. Conserved aa of the In1-ghrelin and In2-ghrelin variants are highlighted in dark grey, aa belonging to the same group are highlighted in light grey, while aa not shared across species are represented in white (not highlighted). 18 of 24 aa of the human, baboon and mouse In1-ghrelin or In2-ghrelin variants are identical or belong to the same group (75% homology). Of note, 22 out of 24 aa are conserved between human and baboon In1-ghrelin variants (92% homology). The novel sequence of the baboon In1-ghrelin variant was submitted to Genbank (accession #: HM048926).
(PPT)

Table S1 **Primers used to identify and clone human In1-ghrelin isoform.**
(PPTX)

Table S2 **Sequences and product sizes of primers used in qrt-PCR.**
(PPTX)

Table S3 **Absolute mRNA copy number of ghrelin axis components in human tissues included in the commercial panel of total RNA from Clontech.**
(PPTX)

Table S4 **Expression level of ghrelin axis in breast tissues and cell lines.**
(PPTX)
