# Supplementary figures and images for: A Novel Human Ghrelin Variant (In1-Ghrelin) and Ghrelin-O-Acyltransferase Are Overexpressed in Breast Cancer: Potential Pathophysiological Relevance
Source: PLoS One. 2011 Aug 4;6(8):e23302. doi: 10.1371/journal.pone.0023302 (PMC3150424; doi:10.1371/journal.pone.0023302)

## Slide 1
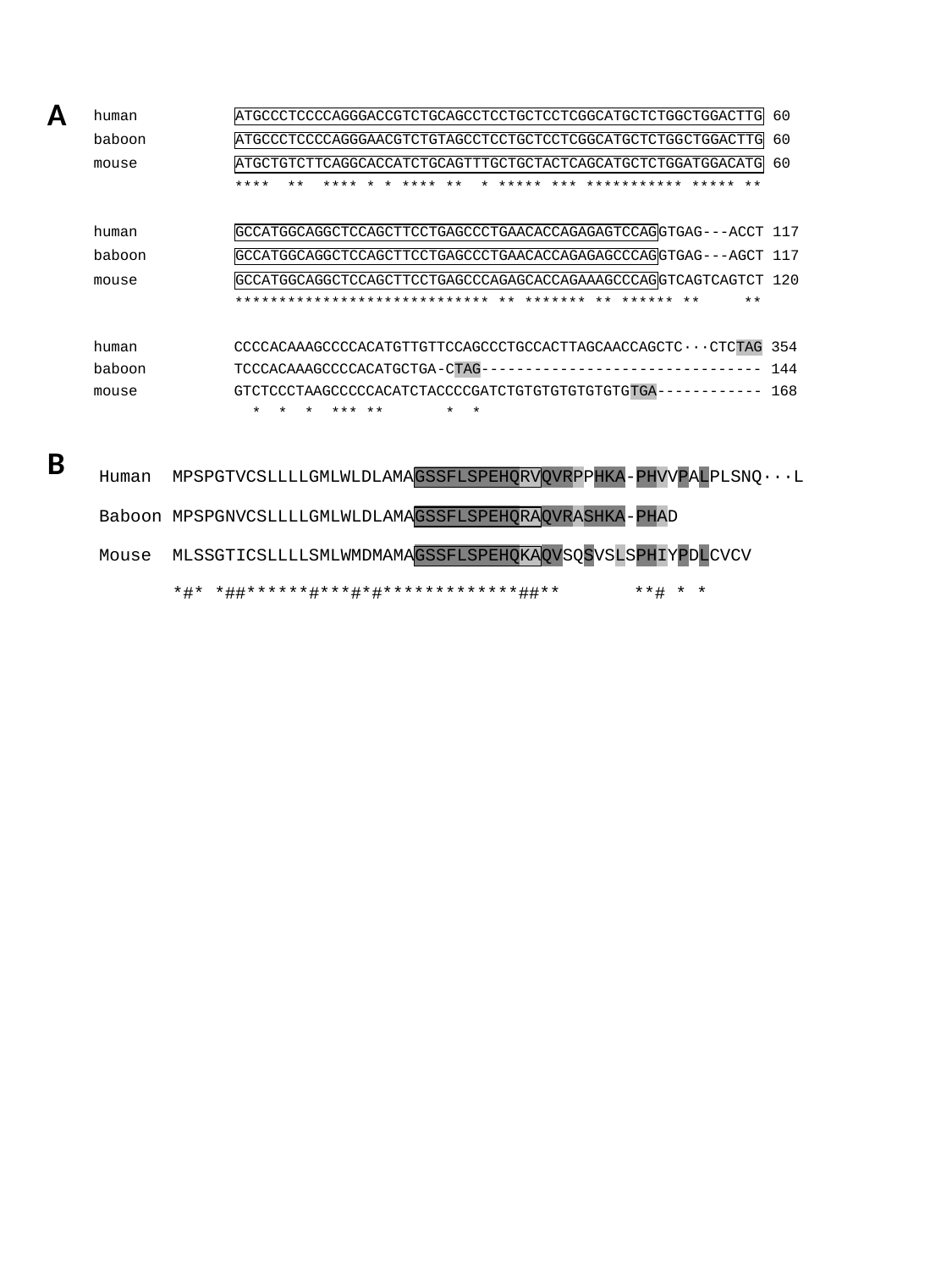

A
B

Supplement: Figure S1 — mRNA and putative protein sequence comparison of human (Homo sapiens) and baboon (Papio anubis) In1-ghrelin variants, and the orthologous mouse (Mus musculus) In2-ghrelin variants. (A) Alignment of the mRNA coding region (CDS) sequences of the In1-ghrelin and In2-ghrelin vaiants. Nucleotide sequences of the Exon(Ex) 1 of the In1-ghrelin and Ex2 of the In2-ghrelin variants that are shared with native-ghrelin are shown in boxes. CDS of baboon In1-ghrelin and mouse In2-ghrelin variants are shorter than human CDS because their mRNAs contain a stop codon (TAG; represented in shaded grey) located in the sequence of In1 or In2, respectively, while the stop codon of the human In1-ghrelin variant is located inside the Ex2 sequence. Therefore, human In1-ghrelin variant mRNA is not fully represented (nucleotides from 172 to 348 are not represented). Asterisks (*) indicate conserved nucleotides across species, which represent a 72% homology among human, baboon and mouse sequences. Of note, the homology between human and baboon In1-ghrelin variant mRNAs is 96%. (B) Alignment of putative In1-ghrein and In2-ghrelin variants protein sequences. Similar to that shown with nucleotide CDS sequences, human In1-ghrelin variant amino acid (aa) sequece is not completely represented (aa from 56 to 116 are not represented). The first 24 aa represents the signal peptide sequences in the three species. Asterisks (*) indicate conserved aa across species (16/24; 67% homology) while (#) indicate aa belonging to the same group (7/24) which results in a 96% homology (23/24 aa) among the human, baboon and mouse sequences. Boxes represent the aa sequence of In1-ghrelin and In2-ghrelin variants shared with native ghrelin. Conserved aa of the In1-ghrelin and In2-ghrelin variants are highlighted in dark grey, aa belonging to the same group are highlighted in light grey, while aa not shared across species are represented in white (not highlighted). 18 of 24 aa of the human, baboon and mouse In1-ghrelin o [file pone.0023302.s001.ppt]
