# Supplementary material for: A Novel Human Ghrelin Variant (In1-Ghrelin) and Ghrelin-O-Acyltransferase Are Overexpressed in Breast Cancer: Potential Pathophysiological Relevance
Source: PLoS One. 2011 Aug 4;6(8):e23302. doi: 10.1371/journal.pone.0023302 (PMC3150424; doi:10.1371/journal.pone.0023302)

## Slide 1
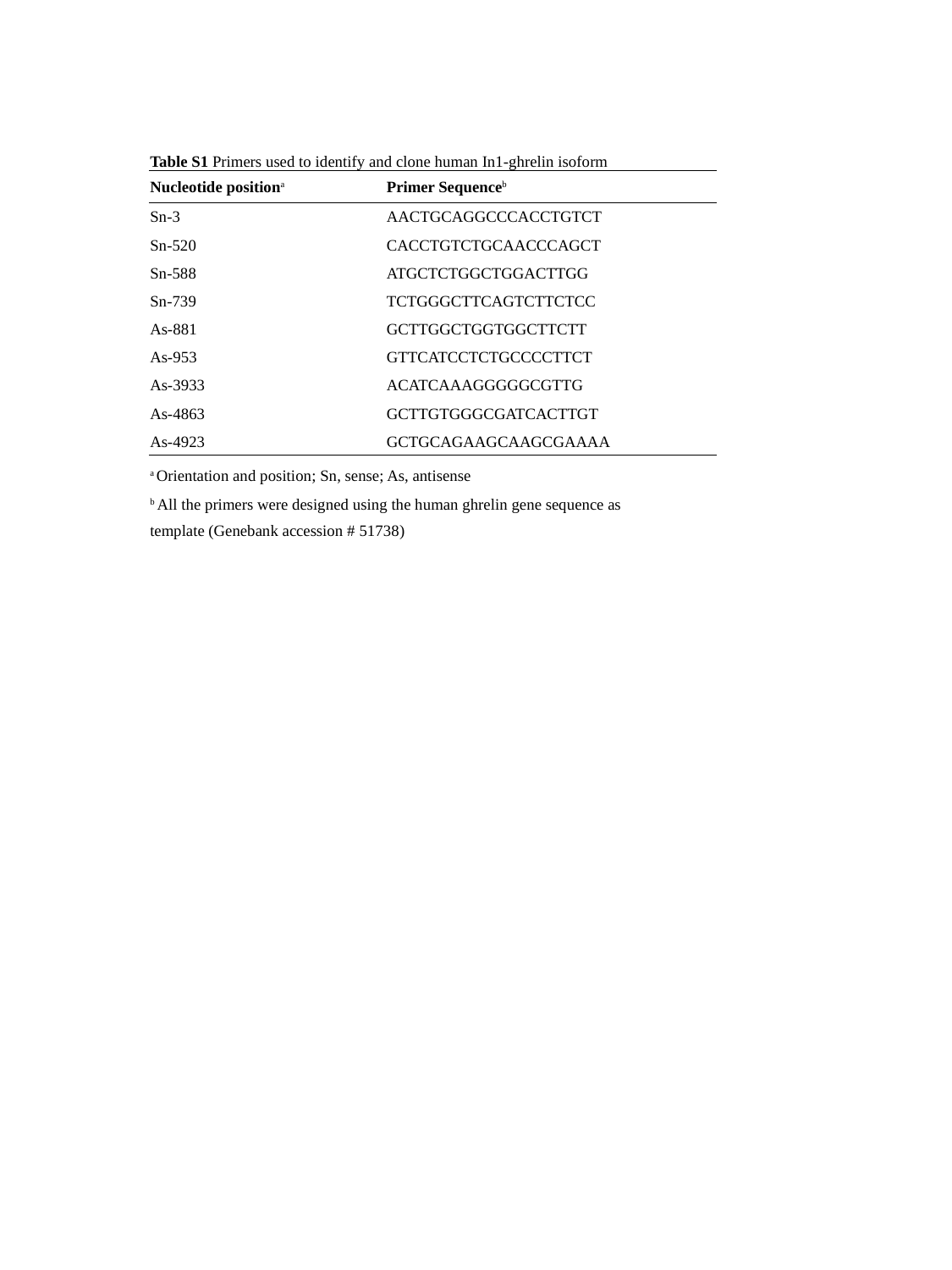

| Table S1 Primers used to identify and clone human In1-ghrelin isoform | |
| --- | --- |
| Nucleotide positiona | Primer Sequenceb |
| Sn-3 | AACTGCAGGCCCACCTGTCT |
| Sn-520 | CACCTGTCTGCAACCCAGCT |
| Sn-588 | ATGCTCTGGCTGGACTTGG |
| Sn-739 | TCTGGGCTTCAGTCTTCTCC |
| As-881 | GCTTGGCTGGTGGCTTCTT |
| As-953 | GTTCATCCTCTGCCCCTTCT |
| As-3933 | ACATCAAAGGGGGCGTTG |
| As-4863 | GCTTGTGGGCGATCACTTGT |
| As-4923 | GCTGCAGAAGCAAGCGAAAA |
| a Orientation and position; Sn, sense; As, antisense | |
| b All the primers were designed using the human ghrelin gene sequence as | |
| template (Genebank accession # 51738) | |

## Slide 2
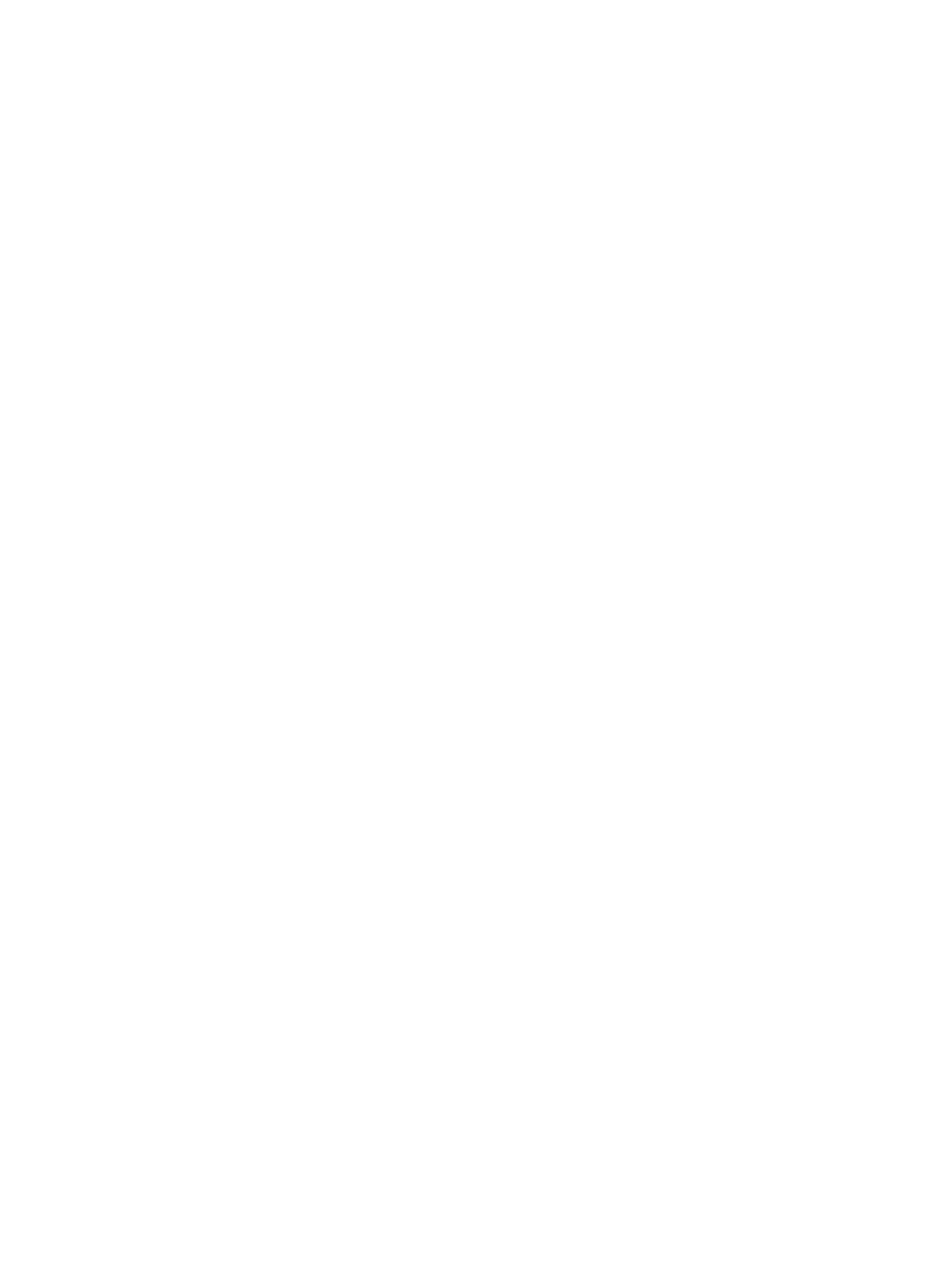

Supplement: Table S1 — Primers used to identify and clone human In1-ghrelin isoform. (PPTX) [file pone.0023302.s002.pptx]
