# Supplementary material for: A Novel Human Ghrelin Variant (In1-Ghrelin) and Ghrelin-O-Acyltransferase Are Overexpressed in Breast Cancer: Potential Pathophysiological Relevance
Source: PLoS One. 2011 Aug 4;6(8):e23302. doi: 10.1371/journal.pone.0023302 (PMC3150424; doi:10.1371/journal.pone.0023302)
Supplement: Table S2 — Sequences and product sizes of primers used in qrt-PCR. (PPTX) [file pone.0023302.s003.pptx]

## Slide 1
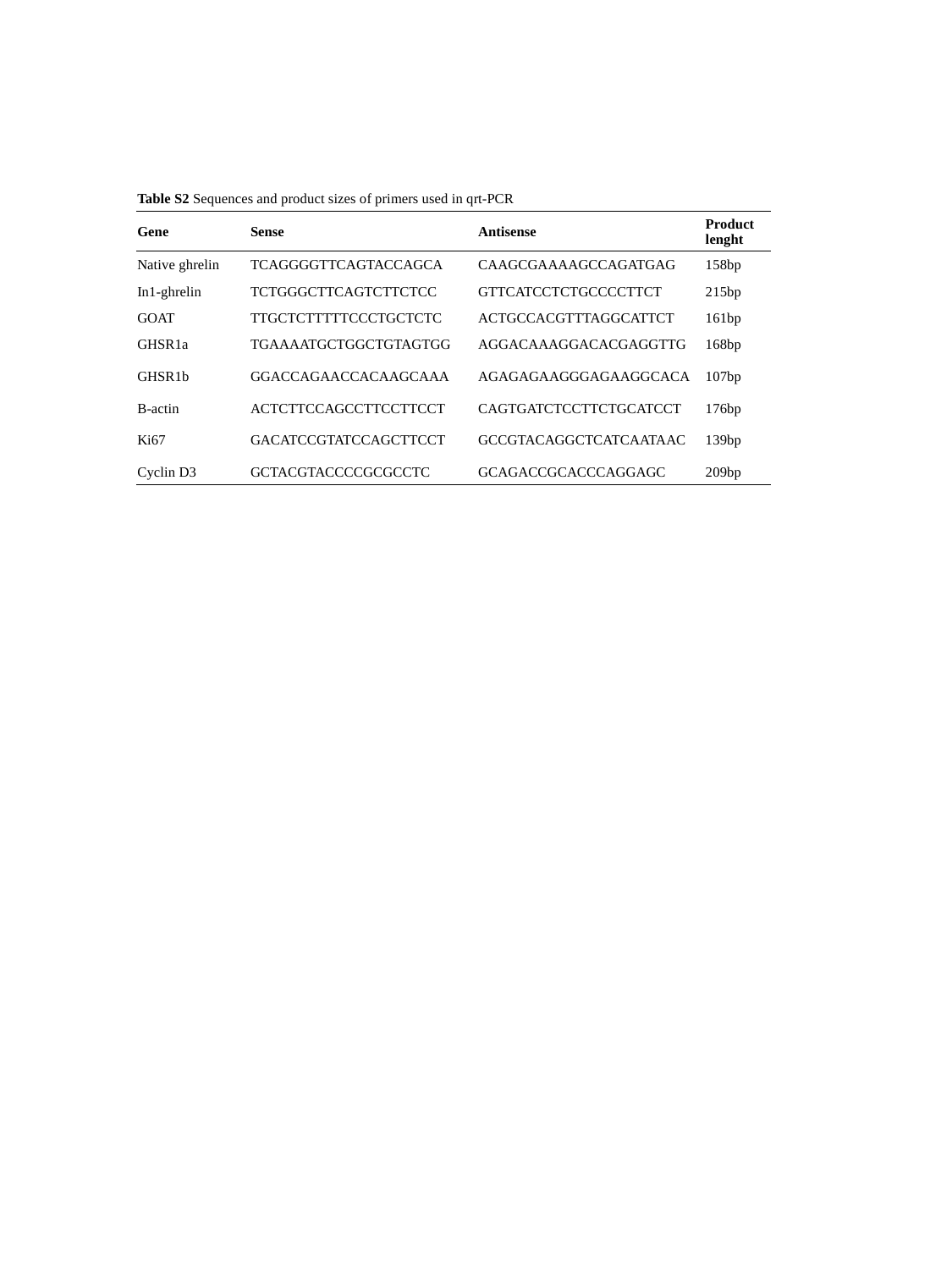

| Table S2 Sequences and product sizes of primers used in qrt-PCR | | | |
| --- | --- | --- | --- |
| Gene | Sense | Antisense | Product lenght |
| Native ghrelin | TCAGGGGTTCAGTACCAGCA | CAAGCGAAAAGCCAGATGAG | 158bp |
| In1-ghrelin | TCTGGGCTTCAGTCTTCTCC | GTTCATCCTCTGCCCCTTCT | 215bp |
| GOAT | TTGCTCTTTTTCCCTGCTCTC | ACTGCCACGTTTAGGCATTCT | 161bp |
| GHSR1a | TGAAAATGCTGGCTGTAGTGG | AGGACAAAGGACACGAGGTTG | 168bp |
| GHSR1b | GGACCAGAACCACAAGCAAA | AGAGAGAAGGGAGAAGGCACA | 107bp |
| B-actin | ACTCTTCCAGCCTTCCTTCCT | CAGTGATCTCCTTCTGCATCCT | 176bp |
| Ki67 | GACATCCGTATCCAGCTTCCT | GCCGTACAGGCTCATCAATAAC | 139bp |
| Cyclin D3 | GCTACGTACCCCGCGCCTC | GCAGACCGCACCCAGGAGC | 209bp |
