# Supplementary material for: A Novel Human Ghrelin Variant (In1-Ghrelin) and Ghrelin-O-Acyltransferase Are Overexpressed in Breast Cancer: Potential Pathophysiological Relevance
Source: PLoS One. 2011 Aug 4;6(8):e23302. doi: 10.1371/journal.pone.0023302 (PMC3150424; doi:10.1371/journal.pone.0023302)
Supplement: Table S3 — Absolute mRNA copy number of ghrelin axis components in human tissues included in the commercial panel of total RNA from Clontech. (PPTX) [file pone.0023302.s004.pptx]

## Slide 1
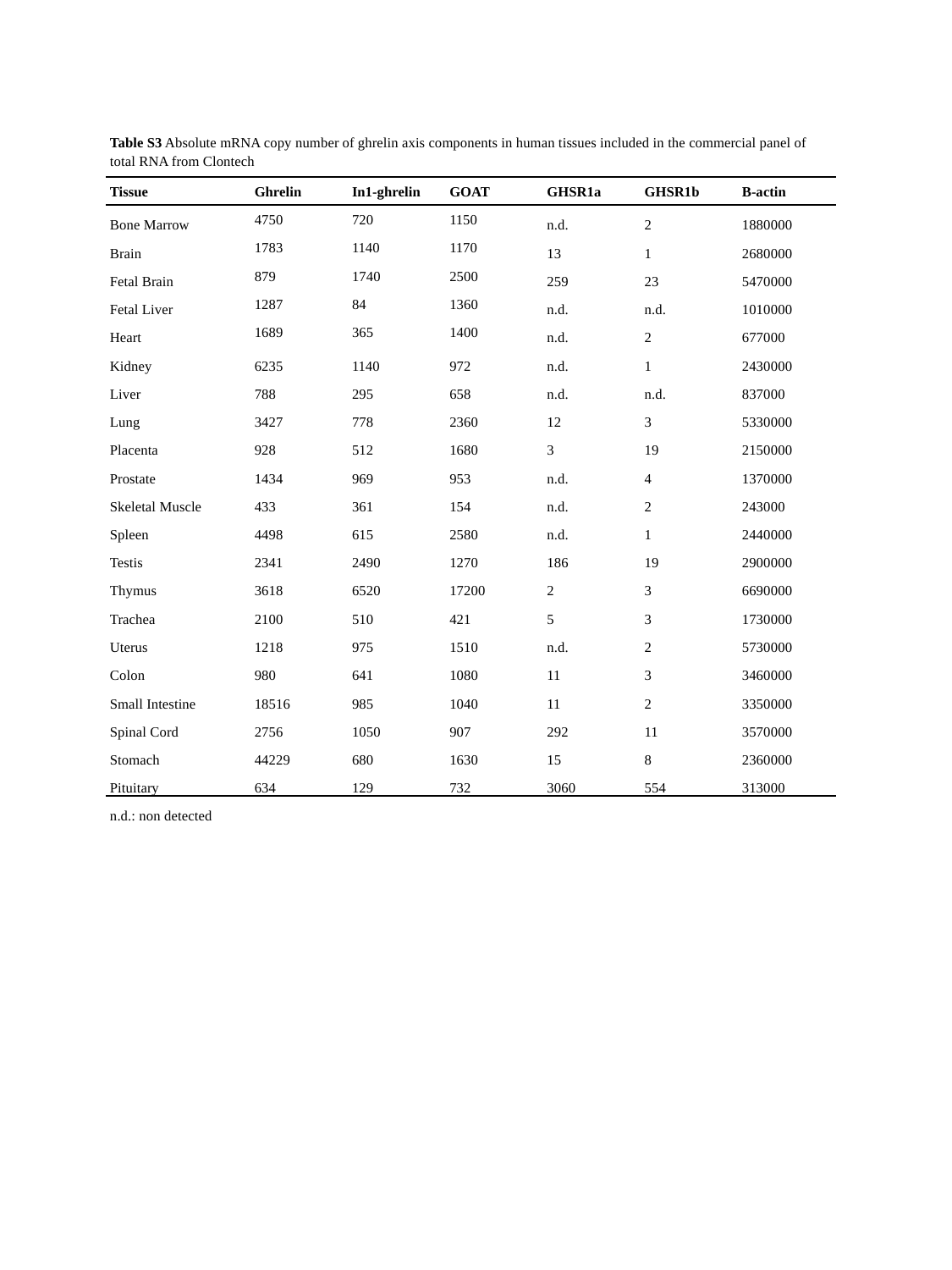

| Table S3 Absolute mRNA copy number of ghrelin axis components in human tissues included in the commercial panel of total RNA from Clontech | | | | | | |
| --- | --- | --- | --- | --- | --- | --- |
| Tissue | Ghrelin | In1-ghrelin | GOAT | GHSR1a | GHSR1b | B-actin |
| Bone Marrow | 4750 | 720 | 1150 | n.d. | 2 | 1880000 |
| Brain | 1783 | 1140 | 1170 | 13 | 1 | 2680000 |
| Fetal Brain | 879 | 1740 | 2500 | 259 | 23 | 5470000 |
| Fetal Liver | 1287 | 84 | 1360 | n.d. | n.d. | 1010000 |
| Heart | 1689 | 365 | 1400 | n.d. | 2 | 677000 |
| Kidney | 6235 | 1140 | 972 | n.d. | 1 | 2430000 |
| Liver | 788 | 295 | 658 | n.d. | n.d. | 837000 |
| Lung | 3427 | 778 | 2360 | 12 | 3 | 5330000 |
| Placenta | 928 | 512 | 1680 | 3 | 19 | 2150000 |
| Prostate | 1434 | 969 | 953 | n.d. | 4 | 1370000 |
| Skeletal Muscle | 433 | 361 | 154 | n.d. | 2 | 243000 |
| Spleen | 4498 | 615 | 2580 | n.d. | 1 | 2440000 |
| Testis | 2341 | 2490 | 1270 | 186 | 19 | 2900000 |
| Thymus | 3618 | 6520 | 17200 | 2 | 3 | 6690000 |
| Trachea | 2100 | 510 | 421 | 5 | 3 | 1730000 |
| Uterus | 1218 | 975 | 1510 | n.d. | 2 | 5730000 |
| Colon | 980 | 641 | 1080 | 11 | 3 | 3460000 |
| Small Intestine | 18516 | 985 | 1040 | 11 | 2 | 3350000 |
| Spinal Cord | 2756 | 1050 | 907 | 292 | 11 | 3570000 |
| Stomach | 44229 | 680 | 1630 | 15 | 8 | 2360000 |
| Pituitary | 634 | 129 | 732 | 3060 | 554 | 313000 |
| n.d.: non detected | | | | | | |
