# Supplementary material for: A Novel Human Ghrelin Variant (In1-Ghrelin) and Ghrelin-O-Acyltransferase Are Overexpressed in Breast Cancer: Potential Pathophysiological Relevance
Source: PLoS One. 2011 Aug 4;6(8):e23302. doi: 10.1371/journal.pone.0023302 (PMC3150424; doi:10.1371/journal.pone.0023302)
Supplement: Table S4 — Expression level of ghrelin axis in breast tissues and cell lines. (PPTX) [file pone.0023302.s005.pptx]

## Slide 1
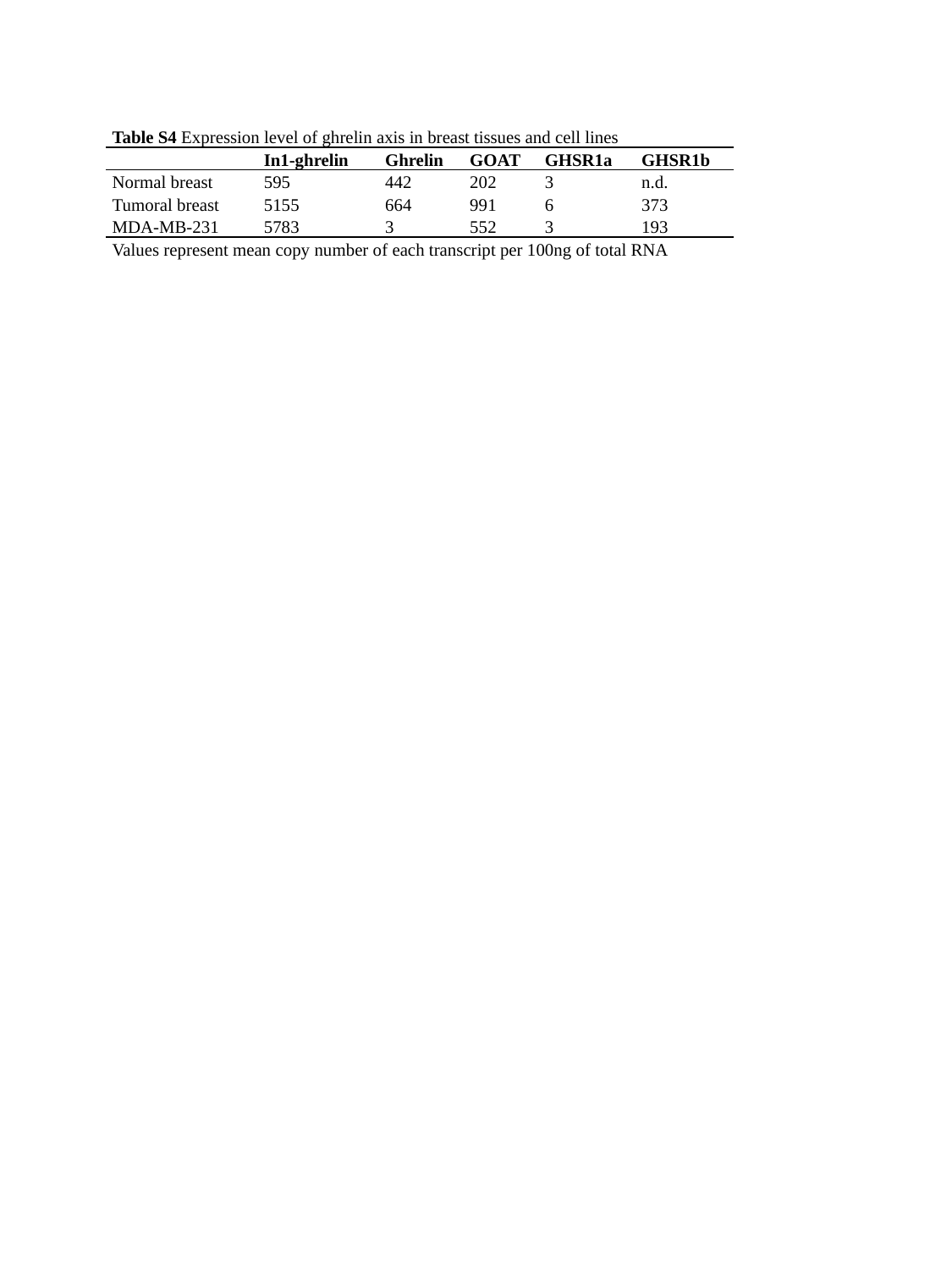

| Table S4 Expression level of ghrelin axis in breast tissues and cell lines | | | | | |
| --- | --- | --- | --- | --- | --- |
| | In1-ghrelin | Ghrelin | GOAT | GHSR1a | GHSR1b |
| Normal breast | 595 | 442 | 202 | 3 | n.d. |
| Tumoral breast | 5155 | 664 | 991 | 6 | 373 |
| MDA-MB-231 | 5783 | 3 | 552 | 3 | 193 |
| Values represent mean copy number of each transcript per 100ng of total RNA | | | | | |
